# On the Use of Arsenic in Eruption

**Published:** 1808-03

**Authors:** W. Weaver

**Affiliations:** Surgeon. Walsall


### *To the Editors of the Medical and Physical Journal.*

GENTLEMEN,

THE ancient physicians, and even kings, employed much time and attention in discovering antidotes to poisons. Poisons appeared to them to be the most formidable diseases, and of course the prevention or cure of them, the most valuable arcanum of the materia medica. Hence we have the grand mithridate, and the various confections of Damocrates, Paulus, &c. &c.

As the ancients, however, knew nothing of chemical agency or chemical preparations, their poisons were limited to the animal and vegetable kingdoms of nature, and of course their antidotes and remedies were derived from the same sources. Chemistry has developed the resources of the mineral kingdom. When Storck of Vienna, and Gataker of London, had taught the profession to respect, instead of dreading, the vegetable poisons; and to consider der them as valuable instruments in the hands of skilful practitioners, Chemistry stepped forward to see what might be done by mineral poisons. Corrosive sublimate (hydrargyrus muriatus,) is still respected; the barytes muriata of Dr. Crawford is gone to the tomb of all the Capulets, merely because it undertook, in all cases, to cure that Protiform disease scrofula, (which is never the same disorder in two patients) by one remedy. The quackish error of extending the virtue of any one remedy too widely, has injured many valuable articles of the materia medica.

There is one chemical preparation from the mineral kingdom that appears to rise in the estimation of medical practitioners, in proportion to their experience of its efficacy; you, Gentlemen, will anticipate my naming it, ARSENIC. And I subscribe cordially to the experience of the numerous London Practitioners, as well as to that of those not less respectable in the country, in prefering Fowler's solution to any other form of exhibiting this powerful and most valuable remedy.

Of all those diseases which have been termed, whether justly or unjustly, the opprobria medicinæ, or medicorum, for there is cause enough to doubt whether the science or the practitioner is in fault; we may reckon gout, cancer, gutta rosacea, noli me tangere, or any other form of a red, pimply, nose or face, may be considered as most distressing to both sexes; and the latter discolourations of the face, are very vexatious to one sex, and generally, tho' erroneously, judged disgraceful to the other. For the following case, as well as a thousand others, will prove that a red, pimply nose, or a Boniface, is no proof of the possessor ever having been addicted to the abuse of spirituous liquors.

I am informed that several physicians in London have administered the oxygenated muriatic acid, or more properly the bleaching liquor, with great success, in those cases where Gowland's dangerous lotion is often used. This practice seems to have originated in an idea that the same preparation which discharges the stains of port or claret from a table cloth, will remove the stains of gin or brandy, or diseased liver, from the face.—But to my Case.

William Lovell, hostler, at the George Inn in this town, made application to me, on account of an eruption of a peculiar kind, not easily defined upon any nosological system; it was universally over his body, but more particularly, the *nose* and *arms*, the former of which had much the appearance of the *gutta rosacea*, and excited suspicion that my patient was a friend or votary of *Bacchus*; such, however, however, was not the case, as his general good character, and abstemious mode of living, evinced the contrary.— His eyes were much affected with chronic inflammation; and, under his *jaws* were large strumous swellings, indicative of scrophula; the eruption upon the arms and body resembled *venereal blotches*, yet, not being produced from that cause, and not so regular a concomitant of scrophula as *scorbutus*.

Considering that the disease might have taken its birth from laxity and debility of the lymphatic or absorbent system, as such I considered the solutio mineralis, a medicine possessing great tonic powers, well adapted for a case depending upon *direct debility*. My patient began by taking eight drops bis die, ex cyath. aquæ, and, in order to preserve a regular state of the bowels, ordered the pilul. plumeri duo om. nocte. He was desired to increase the solution one drop each dose, till unpleasant sensations might forbid a further augmentation; a strict respect was also had to regimen. My patient having no objection, being particularly anxious to have suspicious *Bibo* removed from so exalted and conspicuous a situation as the *nose*, he was consequently very conformable, and persevered in the *solution*, regularly increasing the quantity till he had taken twenty drops each dose, without experiencing any inconvenience; but, on the contrary, the efflorescence had disappeared from the *nose*, which had resumed its former healthy aspect.—The eyes were considerably better; the indurated glands had made a rapid retreat, and the eruption in general, progressively disappearing, his health, which had been very precarious, was improved. From the favourable appearance of things, I thought it prudent to connue the solution, and further increase the dose to twenty-five drops, which, for the first time, occasioned stupor and giddiness; he was therefore directed to take only twenty, till the cure was completed; which happy effect was speedily accomplished.

This *Case*, beyond a doubt, is as decisive in favour of the mineral solution as can possiby come under the observation of a medical man. I am aware, that some may be disposed to scrutinize the propriety of extending the *dose* of so powerful a medicine to the quantity given; should there be such, I am prepared to contend the point, being furnished with materials.

I shall conclude by observing, that a *timid practitioner* is capable of doing *but little good*, and the *bold, daring*, and *assuming emperic, much mischief.*

I am, &c.

*Walsall.* W. WEAVER, Surgeon.

